# A new approach to understanding the impact of circadian disruption on human health

**DOI:** 10.1186/1740-3391-6-7

**Published:** 2008-05-29

**Authors:** Mark S Rea, Andrew Bierman, Mariana G Figueiro, John D Bullough

**Affiliations:** 1 Lighting Research Center, Rensselaer Polytechnic Institute, 21 Union Street, Troy, NY 12180, USA

## Abstract

**Background:**

Light and dark patterns are the major synchronizer of circadian rhythms to the 24-hour solar day. Disruption of circadian rhythms has been associated with a variety of maladies. Ecological studies of human exposures to light are virtually nonexistent, however, making it difficult to determine if, in fact, light-induced circadian disruption directly affects human health.

**Methods:**

A newly developed field measurement device recorded circadian light exposures and activity from day-shift and rotating-shift nurses. Circadian disruption defined in terms of behavioral entrainment was quantified for these two groups using phasor analyses of the circular cross-correlations between light exposure and activity. Circadian disruption also was determined for rats subjected to a consistent 12-hour light/12-hour dark pattern (12L:12D) and ones subjected to a "jet-lagged" schedule.

**Results:**

Day-shift nurses and rats exposed to the consistent light-dark pattern exhibited pronounced similarities in their circular cross-correlation functions and 24-hour phasor representations except for an approximate 12-hour phase difference between species. The phase difference reflects the diurnal versus nocturnal behavior of humans versus rodents. Phase differences within species likely reflect chronotype differences among individuals. Rotating-shift nurses and rats subjected to the "jet-lagged" schedule exhibited significant reductions in phasor magnitudes compared to the day-shift nurses and the 12L:12D rats. The reductions in the 24-hour phasor magnitudes indicate a loss of behavioral entrainment compared to the nurses and the rats with regular light-dark exposure patterns.

**Conclusion:**

This paper provides a quantitative foundation for systematically studying the impact of light-induced circadian disruption in humans and in animal models. Ecological light and activity data are needed to develop the essential insights into circadian entrainment/disruption actually experienced by modern people. These data can now be obtained and analyzed to reveal the interrelationship between actual light exposures and markers of circadian rhythm such as rest-activity patterns, core body temperature, and melatonin synthesis. Moreover, it should now be possible to bridge ecological studies of circadian disruption in humans to parametric studies of the relationships between circadian disruption and health outcomes using animal models.

## Background

As the earth rotates, all species on the surface of the planet are exposed to 24-hour patterns of light and darkness. In response to these regular, daily oscillations to the natural light-dark cycle, these species have evolved endogenous circadian rhythms that repeat approximately every 24 hours [1,2]. Examples of circadian rhythms include oscillations in core body temperature [3], hormone secretion [4], sleep [5], and alertness [6]. Circadian oscillations also exist at a cellular level, including cell mitosis and DNA damage response [7,8]. These oscillations are a result of a small group of clock genes inside the cell nuclei creating interlocked transcriptional and post-translational feedback loops. The timing of these circadian clock genes is generally orchestrated by a master biological clock located in the suprachiasmatic nuclei (SCN) [9] of the hypothalamus of the brain [10]. The master clock in the SCN provides precise time cues throughout the body to regulate these diverse physiological, hormonal, and behavioral circadian patterns. However, in total darkness the timing of the SCN will become asynchronous with the solar day because in humans the period of the master clock is slightly longer than 24 hours [1]. To maintain synchrony with the external world, the light-dark pattern incident on the retina resets the timing of the SCN, so that as we travel across time zones, we can entrain our biological functions to the local environment. If the period of the light-dark pattern is too long or too short, or if the light and dark exposures become aperiodic, the master clock can lose control of the timing of peripheral circadian clocks.

Maintaining the phase-relation ordering of the various circadian rhythms from molecular to behavioral levels appears to be crucial for coordinated functions throughout the human body. Lack of synchrony between the master clock and the peripheral clocks can lead to asynchronies within cells (e.g., cell cycle) and between organ systems (e.g., liver and pancreas). This breakdown in synchrony, as demonstrated most profoundly with jet lag, disrupts sleep [11], digestion [12], and alertness [13]. Chronic disruptions can contribute to cardiovascular anomalies [14] and accelerated cancerous tumour growth [15] in animal models. In humans, epidemiological studies have shown that rotating-shift nurses, who experience a marked lack of synchrony between activity-rest patterns and light-dark cycles (as shown in this report), are at higher risk of having breast cancer compared to day-shift nurses [16]. In fact, the World Health Organization has identified rotating-shift work as a probable cause of cancer [17]. In addition to heightened cancer risks, other disorders have been associated with rotating-shift work, such as diabetes and obesity, suggesting again a role for circadian disruption in the development and progression of diseases [18].

Despite the growing evidence that circadian disruption negatively affects human health [18,19], the logical chain linking light-induced circadian disruption to morbidity and mortality still has not been forged. If the impact of circadian disruption is to be studied with any degree of accuracy, it is important to quantitatively characterize light and dark as it affects the human circadian system because the light-dark pattern is the primary synchronizing stimulus for our circadian system [1]. It is also necessary to quantify the temporal characteristics of circadian light and dark exposures *actually *experienced by people [20]. Without quantification of the actual circadian light and dark exposures experienced by people, it will be difficult to relate the findings from controlled laboratory studies of light-induced circadian disruption in humans to the expected health of any human sub-population, including rotating-shift workers. These actual circadian light and dark exposures in human populations must also be incorporated into parametric studies using animal models as surrogates for particular human diseases or maladies if we are to gain any detailed insight into the role of circadian disruption on human health. Since nocturnal species are used almost exclusively as animal models in this research, a method needs to be established to relate actual circadian light and dark exposures in humans to parametrically controlled exposures of light and dark using these animal models [21].

This paper is concerned with patterns of circadian light and dark as they affect behavioral entrainment and how more sophisticated studies of the relationship between light-induced circadian disruption and human health might be conducted. Here we present original data from the Daysimeter [20], a device for simultaneously recording light-dark and activity-rest data in humans. Significantly, these data reveal relationships between circadian light-dark patterns actually experienced by day-shift and rotating-shift nurses and their own activity-rest patterns. Original data are also presented for two groups of rats, one placed on a 12L:12D pattern of light and dark and the other placed on a 12L:12D pattern of light and dark regularly reversing every 48 hours. We present a novel methodology to quantify circadian entrainment/disruption in both diurnal and nocturnal species, so as to allow researchers to make direct comparisons of circadian entrainment/disruption across species. Attention to circadian entrainment/disruption, rather than to activity alone or to light and dark, per se, makes it possible to circumvent the diurnal-nocturnal conundrum plaguing many comparative studies of light-induced circadian entrainment/disruption using animal models. We found that the circadian entrainment/disruption patterns for day-shift and rotating-shift nurses were remarkably different, but they were remarkably similar to the patterns for two parallel groups of nocturnal rodents. The marked differences in circadian entrainment/disruption patterns within species together with the marked similarities in circadian entrainment/disruption across species, in addition to the new method for quantifying circadian entrainment/disruption, suggest that health-related problems associated with circadian disruption in humans can be parametrically studied using animal models.

## Methods

### Measuring and characterizing circadian entrainment patterns actually experienced by humans

#### Daysimeter

**Figure 1:**
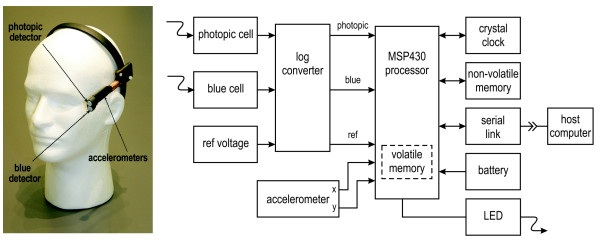
Daysimeter and functional block diagram.

**Figure 2:**
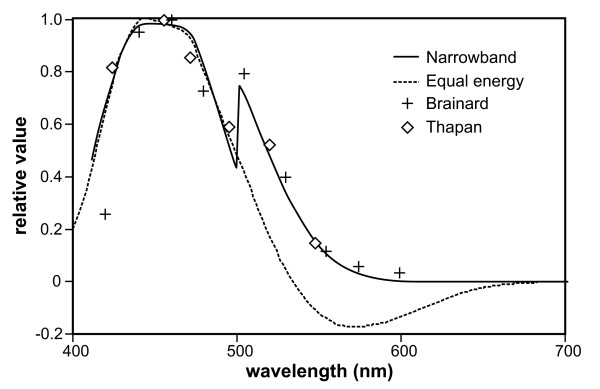
**Spectral response graph**. Spectral response functions generated from the model of human circadian phototransduction by Rea et al. [22]. The dashed line represents the predicted spectral response function for an equal energy spectrum light source. The continuous line represents the predicted spectral responses to individual, narrow-band light sources. The two sets of symbols represent empirical spectral response data from two independent laboratories [34, 35].

**Figure 3:**
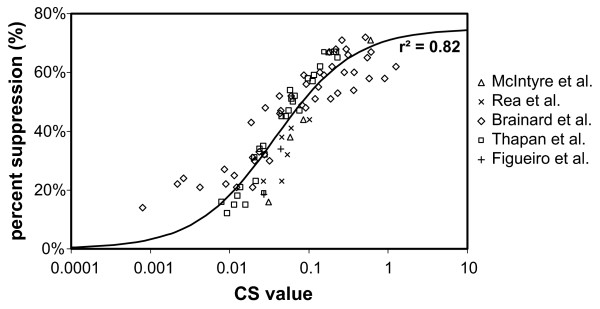
**Logistic transfer function graph**. Logistic transfer function relating nocturnal melatonin suppression to the rectified circadian light stimulus (CS) from the model of human circadian phototransduction by Rea et al. [22]. Data from several studies using both narrow-band [34, 35] and polychromatic light sources [36–38] to induce nocturnal melatonin suppression were plotted as a function of CS. A logistic function from Zeitzer et al. [23] was used to fit the data yielding a regression coefficient (r^2^) for the transfer function equal to 0.82. Figure was adapted from Rea et al. [22].

The Daysimeter was developed as a head-worn light-dosimeter and activity monitor to address measurements of the spectral and spatial response of the human circadian system (Figure 1) [20]. Two detectors are used to characterize the spectral-opponent, subadditive response of the circadian system to polychromatic light and thereby provide measurements of the circadian light stimulus (CS) for humans (Figure 2) [22]. A transfer function relating CS to nocturnal melatonin suppression was also developed [22] to characterize the effective stimulus for non-visual responses associated with optical radiation on the retina (Figure 3). Entrainment to the circadian light-dark pattern is not directly related to nocturnal melatonin suppression, but as demonstrated by Zeitzer et al. [23], both light-induced phase shifting and nocturnal melatonin suppression in humans appear to have similar, if not identical, functional relationships to optical radiation of the same spectral power distribution. The Daysimeter also measures head movements with solid-state accelerometers to characterize behavioral activity. Detailed information about the Daysimeter is available elsewhere [20].

It should be emphasized that activity as measured by the Daysimeter is not a direct measure of the endogenous clock in the SCN. Like every downstream measure of circadian function, behavior can only yield partial insight into circadian entrainment. It is presently impossible to directly measure SCN activity *in vivo*, and thus it is impossible to measure entrainment in the purest sense in living and active humans; the term "behavioral entrainment" is used in this paper to describe the observed levels of synchrony between light-dark exposures and activity-rest responses as measured by the Daysimeter.

#### Data collection

The Daysimeter was sent to nurses throughout the United States to measure their actual CS exposures and activity for seven consecutive days. Forty-three pre-menopausal female nurses, both day-shift (n = 32) and rotating-shift nurses (n = 11), participated in the study. They wore the Daysimeter for seven consecutive days and were scheduled to work at least two and no more than three consecutive days during that period. The Daysimeter was worn while nurses were awake. The nurses were instructed to place the Daysimeter next to them when they slept or bathed. After the seven-day recording session, they returned the device for data analyses. In addition to wearing the Daysimeter, participating nurses provided urine samples, obtained every four hours, for subsequent melatonin assay and filled out a chronotype questionnaire [Horne-Östberg Morningness-Eveningness Questionnaire (MEQ)] and a lighting survey. The nurses were also asked to keep a sleep log, writing down the times they went to bed and any other information about their sleep schedules. These sleep logs were used to match the exact time nurses started wearing the device. Presented here are only the Daysimeter data.

### Measuring and characterizing circadian behavioral entrainment patterns in nocturnal rodents

#### Data collection

Forty albino female Sprague-Dawley rats (*Rattus norvegicus*) were housed in individual cages illuminated by a lighting system previously developed by Bullough et al. [24] to determine the spectral and absolute sensitivities of another nocturnal rodent (*murine*). Based upon the mouse phase response curve (PRC) obtained in that study, a spectral power distribution (nearly monochromatic green light; λ_max _= 525 nm, half-bandwidth = 35 nm) and irradiance (approximately 5 μW/cm^2 ^on the cage floor) were selected to provide the light stimulus to the Sprague-Dawley rats. This particular light stimulus for nocturnal rodents was estimated to be above threshold and below saturation for stimulation of the rat circadian system. The light stimulus for the rats was precisely controlled using a light-emitting diode (LED) light-delivery system fabricated and installed in every cage. The light-delivery system provided better controlled and more biologically meaningful circadian light stimulation to the rats than the fluorescent ceiling lighting traditionally used to provide bright, ambient illumination throughout an animal colony [21].

As with the nurse data, the rat data were obtained from two experimental groups: 20 rats were exposed to a consistently repeating pattern of 12 hours of light (12L) followed by 12 hours of darkness (12D), and another 20 rats (the "jet-lagged" group) were exposed to a 12L:12D pattern where the phase of the light-dark cycle was reversed every 48 hours (as if this group of rats instantly travelled back and forth from Asia to the Americas every other day). Animals were housed individually and allowed to eat and drink *ad libidum*.

Wheel running was measured continuously throughout the experimental session and used as the measure of activity-rest in these animals. The accumulated number of wheel revolutions was recorded at 10-minute time intervals. At the start of the experiment, the photoperiods for both groups were in phase with each other, and the animals exhibited typical nocturnal behavior (active during the dark phase, inactive during the light phase). To allow for acclimation to the cages and to the lighting by the rats, wheel-running data were not collected until the third day of the study, by which time the photoperiod for the "jet-lagged" group had reversed. Most of the activity in the "jet-lagged" group on that day occurred during the light phase. As shown below, the animals in this group were unable to entrain to the regularly reversing photoperiod and exhibited behavior similar to free-running, with similar amounts of activity in the light and in the dark throughout the eight-day observation period.

## Results

**Figure 4:**
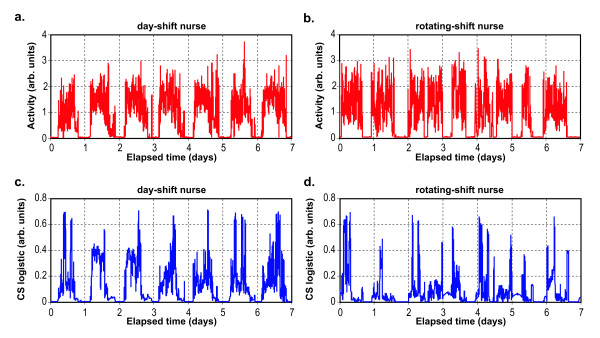
**Activity and light exposure graphs: Nurses**. Activity and light exposure data plotted as a function of elapsed number of days for a day-shift nurse (4a, 4c) and for a rotating-shift nurse (4b, 4d). Data collection started at a different clock time for each subject, so each "day" is a different 24-hour period of time for each subject. Circadian light stimulus (CS) exposures were measured with the Daysimeter [20], and transformed to range between the limits of human melatonin suppression (CS Logistic) shown in Figure 3.

**Figure 5:**
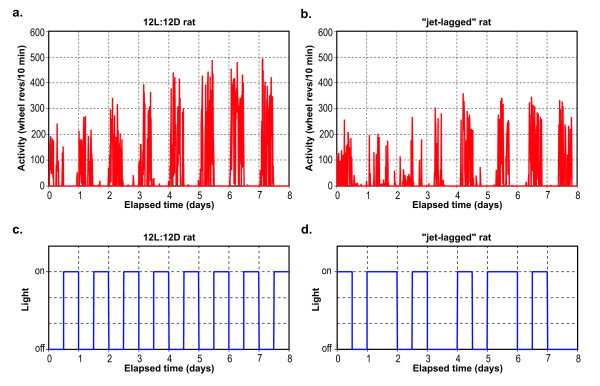
**Activity and light exposure graphs: Rats**. Activity and light exposure data plotted as a function of elapsed time (days) for a 12L:12D rat (5a, 5c) and for a "jet lagged" rat (5b, 5d). At the start of the experiment, the photoperiods were in phase. In the first two days of the experiment, the photoperiods for both groups were the same. Wheel-running data were not collected until the third day of the study, by which time the photoperiod for the "jet-lagged" group had reversed. Most of the activity in the "jet-lagged" group on that day occurred during the light phase.

Figure 4 shows activity and CS exposure data for two representative nurses (one day-shift and one night-shift) and Figure 5 shows the wheel-running data and relative light level for two representative animals (one in the 12L:12D group and one in the "jet-lagged" group).

### Humans

Figures 4a and 4b show activity for two representative nurses, one day-shift nurse (4a) and one rotating-shift nurse (4b), for seven consecutive days. Figures 4c and 4d illustrate the measured CS exposure values obtained directly from the Daysimeter and subsequently transformed using a logistic stimulus-response function representing the entire response range of the circadian system, from threshold to saturation (Figure 3). The transformation was employed to estimate the functional input to the human circadian system, which appears to apply to both light-induced nocturnal melatonin suppression and phase shifting [23].

Examination of Figure 4 reveals subtle but important differences in the activity and transformed CS data for these two nurses. In the case of the day-shift nurse (Figures 4a and 4c), there appears to be a consistent relationship between the activity and transformed CS values over the course of the seven-day measurement session. For the rotating-shift nurse (Figures 4b and 4d), however, this synchrony is much less pronounced. Qualitatively then, and as might be expected, these two example sets of data suggest that the day-shift nurse's behavior is much more synchronized to the light-dark cycle than that of the rotating-shift nurse. Parenthetically, Figure 4 also reveals "flat" periods for both nurses over the course of the seven-day measurement period, which indicate prolonged times of rest and, usually, darkness.

Although many analyses of the activity and of the transformed CS data are possible, the data in Figure 4 were used to develop a quantitative measure of circadian behavioral entrainment/disruption for day-shift and for rotating-shift nurses. The behavioral entrainment analyses were based on the circular cross-correlations of activity and light exposure data. Circular cross-correlation, an analysis technique commonly used in the field of signal processing, involves the concept of time-shifting one signal relative to another to determine relationships between signals that might otherwise be obscured due to relative timing differences. The activity and the transformed CS data can be considered as two time-varying signals whose time-matched values can be multiplied together and then the products at every time of data acquisition integrated into a single value. This value is proportional to the covariance of the two signals. When normalized by dividing by the number of data samples, subtracting the product of the individual signal means, and dividing by the product of the standard deviations of each signal, the result will always be limited to values between -1 and 1 (i.e., a correlation coefficient). The multiply-and-integrate operation can be repeated following a small shift in time by one of the signals (e.g., the activity trace, Figure 4a) with respect to the other (e.g., the transformed CS trace, Figure 4c) and a new correlation coefficient computed. Continuously repeating this process for the entire recording period yields a new time-varying function, the circular cross-correlation, bounded by -1 and 1, that reveals the degree to which the two signals are systematically related to one another for all possible alignments of phase between the two signals. This operation is adapted from standard signal processing techniques [25], and when performed on the periodic light and activity data, yields what are termed, for the purposes of this paper, behavioral entrainment-correlation functions.

**Figure 6:**
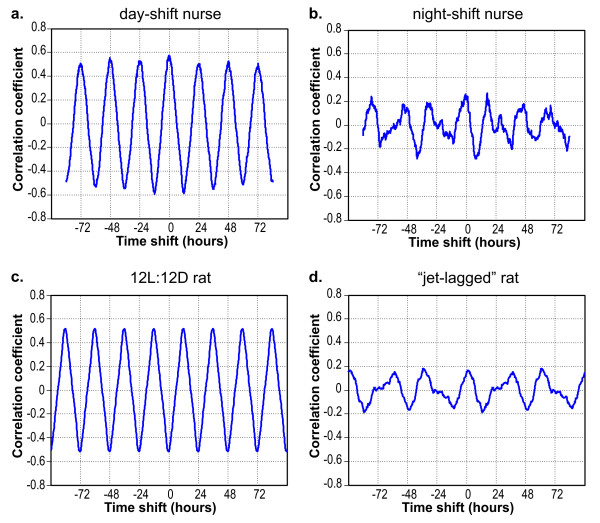
**Behavioral entrainment-correlation functions**. Behavioral entrainment-correlation functions relating activity and light exposures for two example nurses, one day-shift nurse (Figure 6a) and one rotating-shift nurse (6b) and two example rats, a rat exposed to a regular 12L:12D pattern of light and dark (6c) and a "jet-lagged" rat exposed to a 12:12 light-dark cycle that was phase-reversed every 48 hours (6d).

Figure 6 shows two behavioral entrainment-correlation functions relating the transformed CS data to the activity data: one for the day-shift nurse (Figure 6a) and one for the rotating-shift nurse (Figure 6b) in Figure 4. As can be readily appreciated from Figure 6a, the activity of the day-shift nurse is highly entrained to her light-dark pattern throughout the seven days, as exhibited by the regularly oscillating, 24-hour period of her behavioral entrainment-correlation function. More specifically, this nurse, typical of almost all day-shift nurses, has a peak correlation near the zero-phase marker and again at every 24-hour multiple. This day-shift pattern is in marked contrast to the behavioral entrainment-correlation pattern for the rotating-shift nurse (Figure 6b). Her pattern is aperiodic, exhibiting minor correlation peaks at times other than at the 24-hour phase markers. The pattern of the rotating-shift nurse is of much lower amplitude and very distorted compared to the smoothly varying and periodic behavioral entrainment-correlation pattern of the day-shift nurse.

### Nocturnal rodents

The wheel running data from the 12L:12D (e.g., see Figure 5a) rats were typical of those collected in innumerable studies, with more active periods associated with darkness and less active periods in the light. The "jet-lagged" group differed considerably from the 12L:12D group, however, in the apparent degree of association between the light-dark and the rest-activity data (e.g., see Figure 5b). For those rats in the 12L:12D group, almost all of their wheel running occurred in darkness; although, as is usually the case, there was some activity in the light, particularly near the transition times from light to dark, and there were intervals of quiescence sporadically occurring during the dark periods. In the "jet-lagged" group of rats, the association between wheel running and darkness was markedly less pronounced. Indeed, after several reversals of the light-dark cycle, the wheel running appeared to be disassociated with either light or dark.

The same analyses performed on the data from the nurses were also applied to the data from the two groups of nocturnal rodents. The light exposure values were binary for the rats, zero when no cage lighting was present and a value of one when the cage lighting was administered. A behavioral entrainment-correlation function from one typical rat in the 12L:12D group is shown in Figure 6c. The similarity between the entrainment-correlation function for the sample day-shift nurse and the 12L:12D rat are remarkable; the only apparent difference is that the latter function is shifted approximately 12 hours with respect to the former. This shift reflects the expected difference between a diurnal and a nocturnal species; diurnal nurses are active during the day and inactive at night, whereas nocturnal rats are inactive during the light phase and active in the dark. Figure 6d shows a typical behavioral entrainment-correlation function for one rat in the "jet-lagged" group. Again, there is a marked similarity between the entrainment-correlation functions for the rotating-shift nurse in Figure 6b and for the "jet-lagged" rat in Figure 6d.

### Phasor representations of circadian behavioral entrainment

Plots of the behavioral entrainment-correlation functions for the day-shift nurses generally exhibit smooth, oscillating curves whereas those of the rotating-shift nurses exhibit much more irregular patterns. Estimates of the relationship between activity-rest and light-dark in terms of magnitude and phase can be determined for both groups of nurses through Fourier decomposition and spectral analysis of the behavioral entrainment-correlation functions. Phasors represent the magnitude and phase relationship between the activity-rest data and the light-dark data that underlie the entrainment-correlation functions for a particular spectral component obtained from the Fourier decomposition [26]. Since the 24-hour spectral component is of special interest in studies of circadian entrainment, the activity and light data for every nurse were first parsed into seven equal 24-hour periods. The behavioral entrainment-correlation functions were then calculated for each of these seven periods after which the seven corresponding phasors representing the frequency component corresponding to a 24-hour periodicity, f(24) for every one of the 43 nurses were determined. It should be noted that a systematic investigation of periods ranging from 22 to 26 hours in 10-minute increments was conducted for the day-shift nurse data. While the range of peak phasor amplitudes occurred for periods ranging from 23.7 to 24.56 hours as determined from quadratic curve-fits to the phasor magnitude versus period data, the mean was 24.035 hours, supporting the significance of the 24-hour period for this analysis.

**Figure 7:**
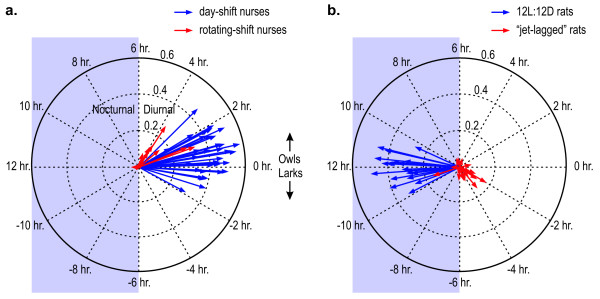
Phasor diagrams for day-shift and rotating-shift nurses and for 12L:12D and "jet-lagged" nocturnal rats.

Complex arithmetic [27] was then used to determine the average (n = 7) phasor for a given nurse and these average phasors for all the nurses are plotted in Figure 7a in polar coordinates. The length of each phasor is the magnitude of the average f(24) and reveals how well light and activity are correlated over the seven-day recording session. As a group, the day-shift nurses have larger phasor lengths than the rotating-shift nurses, implying that they have a much higher degree of behavioral entrainment.

Consistent with a diurnal species, all the phasor directions for the nurses are to the right, meaning that activity and light exposure occur at nearly the same time. The angular direction of a phasor indicates the phase relationship between light and activity for an individual. Greater amounts of activity near the onset of circadian light exposure than near the offset of circadian light exposure produces a phasor extending below the zero-phase polar axis line (labeled 0 hour). Conversely, greater amounts of activity near the offset of circadian light exposure than near the onset of circadian light exposure produces a phasor extending above the zero-phase line. Researchers [28] have used the terms "larks" and "owls" to refer to people with diurnal activity patterns biased toward morning or evening hours, respectively. These times, however, are not explicitly linked to actual light exposures. The phasor analysis does reveal similar behavioral characteristics, but ones referenced to actual light-dark exposures rather than to an arbitrary exogenous time reference (watch or wall-clock time). Borrowing the lark and owl terminology for describing the behavioral characteristics revealed by the phasor analyses, it is interesting to note that there are more owls than larks, particularly among the rotating-shift nurses, indicating that these people tend to be more active after the onset and subsidence of daily light exposure than before. Although it was true that for day-shift nurses the natural solar cycle was largely coincident with the measured light-dark pattern, the phasor analyses are, again, performed without respect to any exogenous time reference. Theoretically then, a person exhibiting lark or owl behavior with respect to actual light and dark pattern could, in fact, be completely out of phase with the local solar day, as indeed would happen with a "true" night-shift worker.

The rats exposed to the consistent 12L:12D light-dark cycle produced average (n = 8) phasors with magnitudes similar to the day-shift nurses, but with directions to the left, clustered around a 12-hour phase shift between light and activity, as would be expected for an entrained nocturnal animal (Figure 7b). The "jet-lagged" rats experiencing the continually changing light-dark exposures have short, low magnitude average (n = 8) phasors with no consistent direction across individuals. (Two very different scenarios can result in the same low magnitude average phasors. One is that every phasor comprising the average is low in magnitude, which indicates that there is no systematic relationship between activity-rest and light-dark. The second, as exhibited by the "jet-lagged" rats, is that individual phasors representing 24-hour periods have significant magnitudes, but their phase varies widely in many directions resulting in a small magnitude average phasor. Either scenario, however, indicates low entrainment to the light-dark pattern when measured across multiple days.)

**Figure 8:**
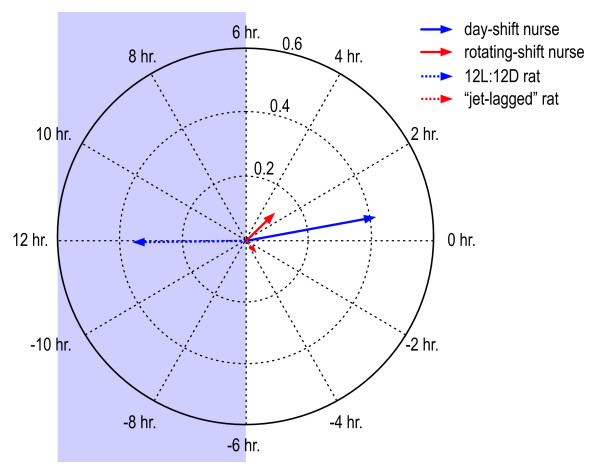
Mean phasors for nurses and for rats.

Figure 8 shows the average [27] phasor magnitudes and phase angles for the two groups of nurses and the two groups of rats. The common use of binary light-dark exposure levels and of wheel running as a measure of activity in caged animals can potentially affect the comparison of their phasor magnitudes to those obtained by humans using the Daysimeter. In a natural environment human activity varies continuously as does a person's light exposure. The phasor analysis based upon the Daysimeter data captures the association between the natural and continuously varying stimuli and responses. Conversely, caged animals have many fewer options with regard to self-regulated light exposures and with regard to running behavior. This situational difference between species may, in fact, have contributed to the relatively shorter phasor magnitudes in the 12L:12D group of rats than in the day-shift nurses. Clearly if cross-species comparisons are to be made, additional investigations need to be undertaken of actual light exposures and of alternative behavioral measures for *both *human and animal models.

### Phasors compared to other measures of circadian behavioral entrainment

**Figure 9:**
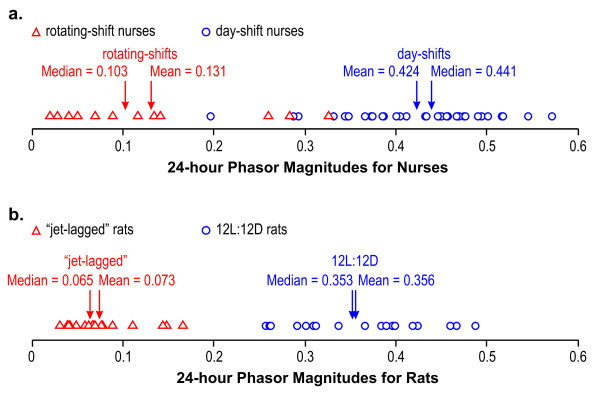
Phasor magnitudes for the day-shift, and rotating-shift nurses (a) and for the two groups of rats (b).

Considering only the degree of behavioral entrainment, Figure 9 shows the distribution of the f(24) phasor magnitudes for the two groups of nurses (Figure 9a) and for the two groups of rats (Figure 9b). Figure 9a shows a clear and statistically significant difference between the day-shift and rotating-shift nurse groups with widely separated group means and medians. Nevertheless, there is some overlap of the distributions, perhaps reflecting a true continuum of the degree of circadian behavioral-entrainment among individuals. The data from the rats in Figure 9b also show a clear and statistically significant separation, but undoubtedly because of the two radically different light-dark patterns, there is no overlap in the phasor amplitudes for these two groups of rats.

The interdaily stability (IS) and the intradaily variability (IV) statistics [29] have been used in numerous studies as measures of behavioral entrainment, or more precisely the coupling between rest-activity rhythms and assumed exogenous *zeitgebers*, or time givers [30-32]. Unlike the phasor analysis, these two statistics are computed based solely on activity and cannot be used to assess the phase relationship between measured activity and the actual light *zeitgeber*.

**Figure 10:**
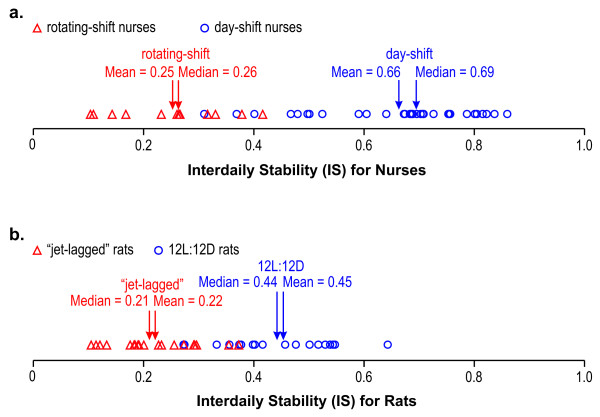
Interdaily stability (IS) statistics for the day-shift and rotating-shift nurses (a) and for the two groups of rats (b).

It is possible, however, to compare phasor magnitudes (Figure 9) and IS values by using the same sets of activity data as estimates of circadian entrainment. The distribution of the IS statistic was calculated from the activity data from nurses (Figure 10a) and from rats (Figure 10b). The two groups of nurses and the two groups of rats were significantly different in terms of their IS values. The ratio of the mean IS values for the two groups of nurses (2.6) and the ratio of the mean IS values for the two groups of rats (2.0) are similar to, but smaller than the ratios of the mean phasor magnitudes for the comparable groups shown in Figure 9 (3.2 for nurses and 4.9 for rats). This comparison between phasor magnitude ratios and IS value ratios suggests that a better assessment of behavioral entrainment can be made by relating measured activity-rest to actual light-dark exposures than to an exogenous time reference, such as local solar time, that may or may not be correlated with the actual *zeitgeber *for entrainment, that is, light.

The IV statistic was also calculated from the activity data from nurses and rats, but the values showed no significant difference between the two groups of nurses nor between the two groups of rats; the mean IV values for day-shift and rotating-shift nurses were 0.50 and 0.54 respectively with standard deviations of 0.20 and 0.16 respectively, and the mean values for the entrained and "jet-lagged" rats were 1.10 and 1.21 with standard deviations of 0.28 and 0.27 respectively. This lack of separation in IV values for the two groups of nurses and for the two groups of rats suggests that consolidation of activity patterns is not systematically related to the degree of circadian behavioral entrainment as measured either with IS values or with phasor magnitudes.

## Discussion

This paper provides a new framework for the study of the effects of circadian entrainment/disruption on human health, emphasizing three important links in the logical chain relating circadian disruption to maladies such as breast cancer, obesity, and sleep disorders [18].

First, circadian light (and dark) for humans and for animal models can now be quantitatively defined to such a degree that meaningful studies of light as a stimulus for circadian disruption can be undertaken, not only in humans but in nocturnal rodents as well. Without quantitative definitions of the light stimuli, it would simply be impossible to understand the results of any ecological study of circadian disruption on human health or how laboratory studies using animal models relate to the human condition. Second, with an understanding of circadian light, it is now possible to measure the synchrony between light-dark and activity-rest patterns in actual human living environments using tools like the Daysimeter [20]. These ecological light and activity data are necessary to develop the essential insights into circadian disruption actually experienced by modern people. Third, it is now possible to simply and quantitatively characterize degrees of circadian entrainment/disruption; that is, the levels of synchrony between light-dark exposures and activity-rest, in both humans and animal models. A focus on entrainment, rather than light per se or activity alone, makes it possible to relate ecological studies of diurnal humans to parametric studies of diseases using nocturnal animal models. In other words, parametric studies of circadian disruption employing animal models for human diseases can now be designed and conducted so as to more accurately reflect their relevance to the actual living conditions in humans.

It should be emphasized, too, that the methods presented here are not limited to the study of behavioral entrainment. Rather, this analysis provides the basis for assessing entrainment of other outcome measures from the circadian system, such as core body temperature or melatonin synthesis, to light-dark patterns. From these envisioned studies, modern maladies like diabetes, obesity, and poor sleep, as well as breast cancer and cardiovascular disease, can be meaningfully and systematically investigated. More important perhaps, forging the links identified in this paper will significantly accelerate a deeper understanding of the role of circadian disruption on human health [17] and thereby may accelerate medical treatment of these maladies with light and with drugs [33]. The techniques identified here also imply that, in the future, it will be possible to examine circadian entrainment/disruption on an individual basis so that each person can be treated with the appropriate light-dark exposure and/or with the appropriate pharmaceutical interventions.

## Competing interests

The authors declare that they have no competing interests.

## Authors' contributions

MSR conceived the study, lead the team in its execution and drafted major sections of the paper, AB formulated the analyses and drafted portions of the Results and Discussion sections, MGF was instrumental in acquiring the nurse data, drafted sections of the paper and provided expertise while preparing the manuscript, JDB was instrumental in acquiring the rat data and provided expertise while preparing the manuscript. All authors participated equally in discussions and the exchange of ideas during the study, and all reviewed and approved the final manuscript.
